# Generating Artificial Patients With Reliable Clinical Characteristics Using a Geometry-Based Variational Autoencoder: Proof-of-Concept Feasibility Study

**DOI:** 10.2196/63130

**Published:** 2025-04-17

**Authors:** Fabrice Ferré, Stéphanie Allassonnière, Clément Chadebec, Vincent Minville

**Affiliations:** 1 Department of Anesthesia, Intensive Care and Perioperative Medicine Purpan University Hospital Toulouse France; 2 Université Paris Cité, Unité Mixte de Recherche S1138, Institut national de recherche en sciences et technologies du numérique, Sorbonne University Paris France

**Keywords:** digital health, artificial data, variational autoencoder, data science, artificial intelligence, health monitoring, deep learning, medical imaging, imaging, magnetic resonance imaging, Alzheimer disease, anesthesia, prediction, data augmentation

## Abstract

**Background:**

Artificial patient technology could transform health care by accelerating diagnosis, treatment, and mapping clinical pathways. Deep learning methods for generating artificial data in health care include data augmentation by variational autoencoders (VAE) technology.

**Objective:**

We aimed to test the feasibility of generating artificial patients with reliable clinical characteristics by using a geometry-based VAE applied, for the first time, on high-dimension, low-sample-size tabular data.

**Methods:**

Clinical tabular data were extracted from 521 real patients of the “MAX” digital conversational agent (BOTdesign) created for preparing patients for anesthesia. A 3-stage methodological approach was implemented to generate up to 10,000 artificial patients: training the model and generating artificial data, assessing the consistency and confidentiality of artificial data, and validating the plausibility of the newly created artificial patients.

**Results:**

We demonstrated the feasibility of applying the VAE technique to tabular data to generate large artificial patient cohorts with high consistency (fidelity scores>94%). Moreover, artificial patients could not be matched with real patients (filter similarity scores>99%, κ coefficients of agreement<0.2), thus guaranteeing the essential ethical concern of confidentiality.

**Conclusions:**

This proof-of-concept study has demonstrated our ability to augment real tabular data to generate artificial patients. These promising results make it possible to envisage in silico trials carried out on large cohorts of artificial patients, thereby overcoming the pitfalls usually encountered in in vivo trials. Further studies integrating longitudinal dynamics are needed to map patient trajectories.

## Introduction

With the growing impact of data science technologies, novel health care ecosystems centered around artificial patients are developing. The data science–based approach for generating artificial patients involves augmenting real data [1]. Thus, new artificial data are created with characteristics similar to those of the original population of interest. Such data could be particularly valuable in clinical research, offering the potential for studies that are not only more cost-effective but, more importantly, also more inclusive and impactful—especially in cases where patient recruitment poses a challenge [2].

From a methodological point of view, artificial labeled data are generated using mechanistic or statistical methods. The mechanistic approach combines known equations from physical, biological, or other fields to describe a phenomenon, referred to as digital twins. This approach could facilitate personalized therapeutics [3], though its routine use in medical processes remains limited [4]. Deep learning methods for generating artificial data in health care include technologies based on generative adversarial networks (GANs) [5] or variational autoencoders (VAEs) [1,6]. However, most of the studies using GANs focused on a fairly large training set (over 1000 training samples) or on low-dimension data, while it remains very challenging to gather such large cohorts of labeled patients. Therefore, the case of high-dimensional data combined with a small sample size (a situation commonly encountered in medicine) remains largely unexplored by GAN technologies. Chadebec et al [1] have recently demonstrated, by using a VAE, that the artificial augmentation of medical imaging data significantly improved classification accuracy. The balanced accuracy increases from 66% to 74% for a convolutional neural network classifier trained with small datasets (50 magnetic resonance images each of cognitively healthy individuals and patients with Alzheimer disease), while improving greatly the sensitivity and specificity of the classification metrics [1]. In other words, a geometry-based VAE was able to produce meaningful samples from high-dimension, low-sample-size (HDLSS) imaging datasets. This method, validated for an image classification task (voxels), deserves to be tested on (clinical) tabular data. Given the multiplicity and complexity of the data obtained in anesthesia, this statistical approach using artificially augmented data could be of major interest to identify predictive clinical factors of poor outcomes with accuracy and reliability. Thus, the aim of our study was to test the feasibility of generating artificial patients with reliable clinical characteristics by using a VAE applied on HDLSS tabular data.

## Methods

### Overview

Clinical tabular data were extracted from 521 real patients of the MAX (BOTdesign) database. MAX is a digital conversational agent for preparing patients for anesthesia [7,8]. Collected data included demographic characteristics, past medical history, medication, and other relevant medical items.

**Figure 1:**
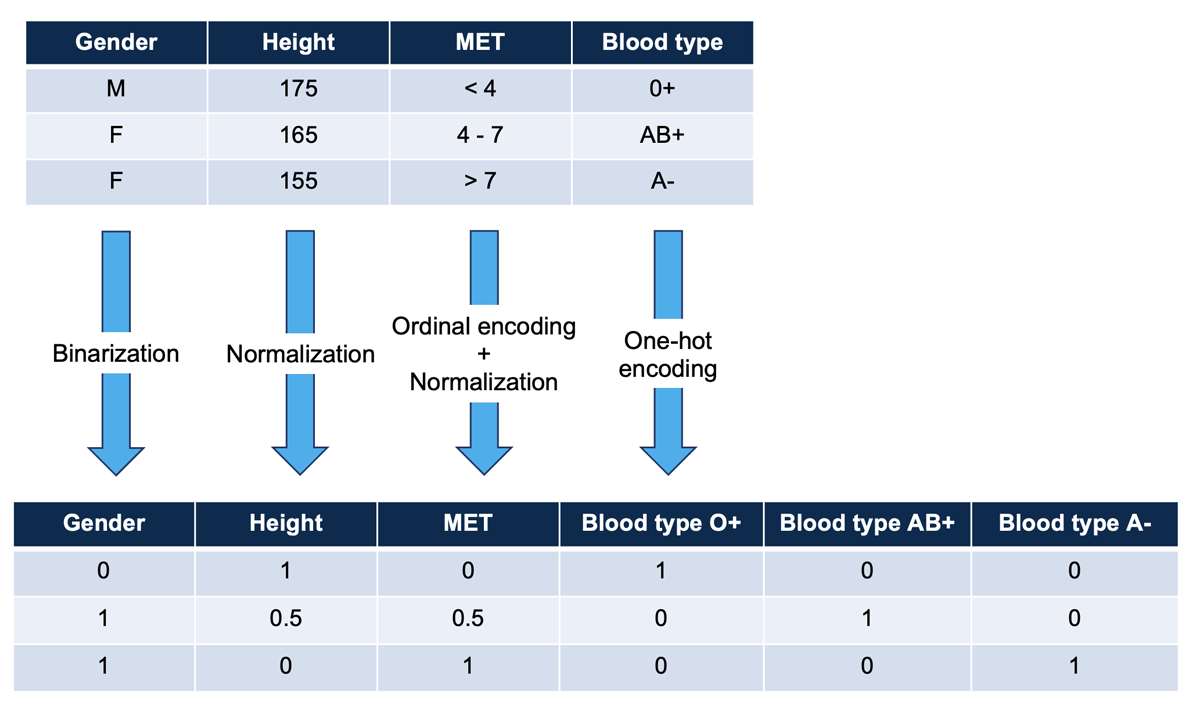
Illustration of the data preprocessing step. MET: metabolic equivalent task.

**Figure 2:**
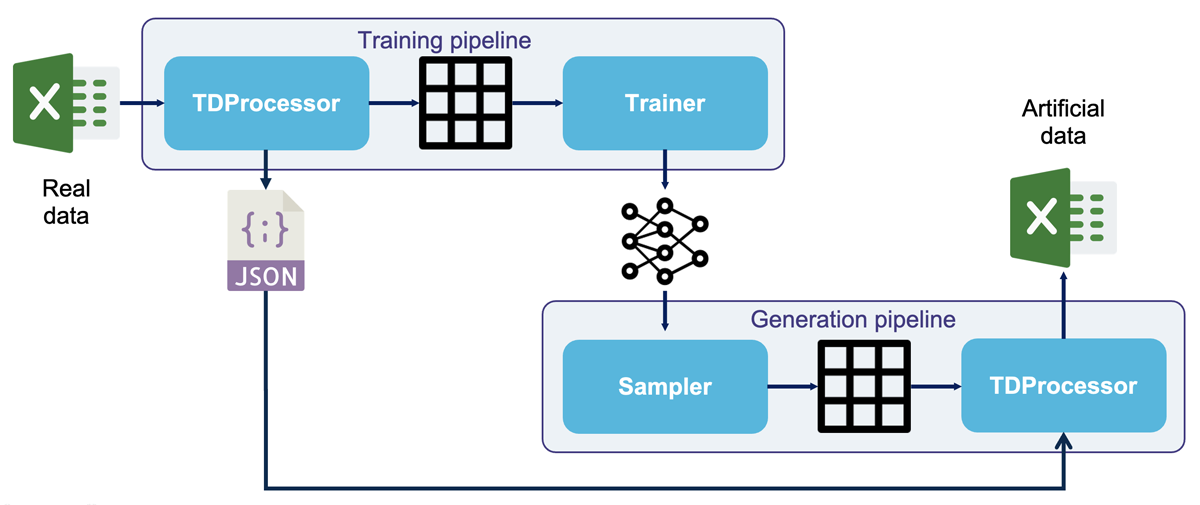
Illustration of the complete pipeline for generating artificial patients. For more details, see Multimedia Appendix 1. TDProcessor: TabularDataProcessor.

Details of the methodological approach used to train the model and generate artificial data are available in Multimedia Appendix 1. Briefly, the dataset included 521 patients about to undergo anesthesia, each with 85 clinical features. Once data preprocessing was completed (Figure 1), the model was trained on a (521, 103) dimension dataset using a modified Pyraug’s training pipeline (Figure 2). Training hyperparameters were set to 1000 epochs, a batch size of 32, and a learning rate of 0.001. Two datasets of 5000 and 10,000 artificial patients were generated, representing a data increase rate of 10 and 20 artificial patients, respectively, for 1 real patient.

The next step involved assessing the consistency (fidelity scores) and confidentiality (filter similarity scores and degree of anonymization) of artificial data.

Finally, the plausibility of newly generated artificial patients was validated through expert human supervision (categorization task).

### Ethical Considerations

We did not seek ethics approval in accordance with the Commision Nationale Informatique et Libertés policy on secondary analyses of preexisting datasets (Titles I and II) [9], as patients were informed that their data may be securely stored, coded for confidentiality, and used for research unless they explicitly object.

## Results

### Consistency and Confidentiality of Artificial Data

**Figure 3:**
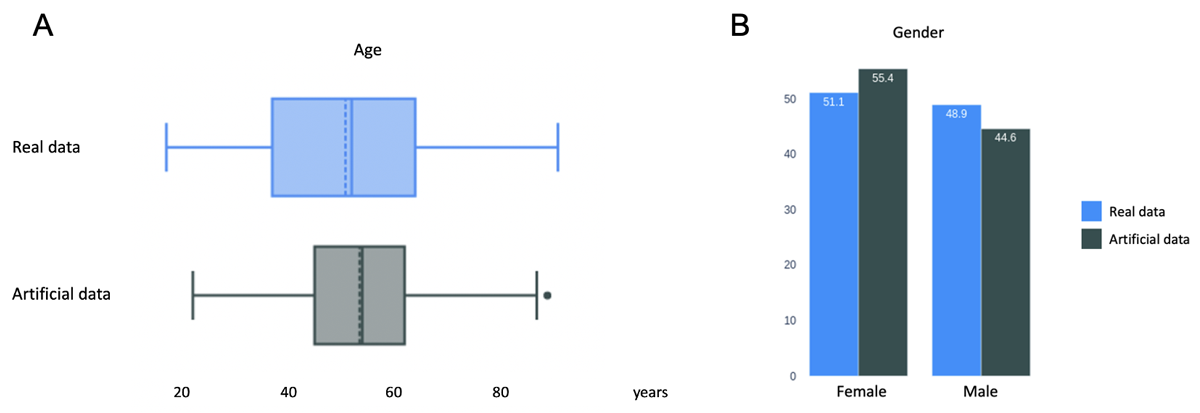
Distribution of (A) the numerical variable “age” and (B) the categorical variable “gender” (in relative percentage) from the datasets of 521 real and 5000 artificial patients.

**Figure 4:**
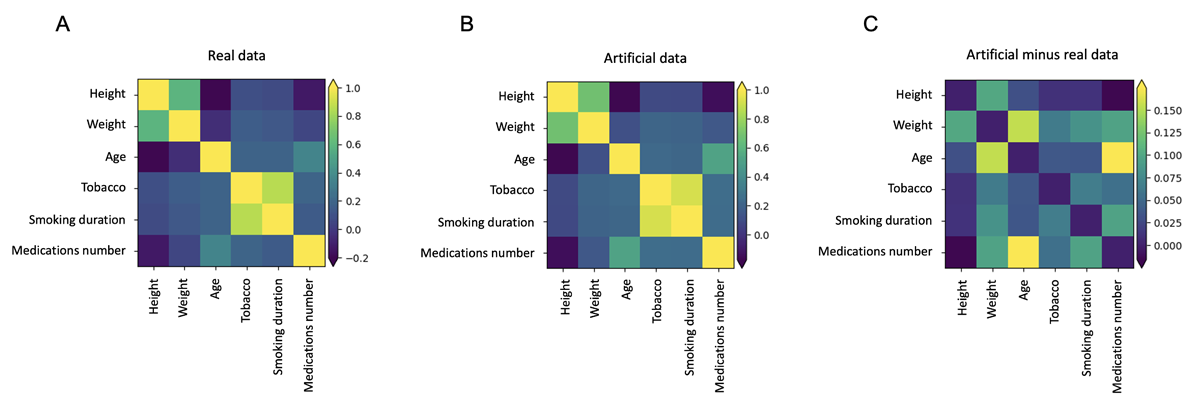
Matrices illustrating the correlation of numerical variables from the datasets of 521 real and 5000 artificial patients (A) and (B) and their differences (C).

To assess the consistency of newly generated artificial data, fidelity scores were calculated. A fidelity score is defined as the arithmetic mean of its 3 components: numerical and categorical data distribution stability and numerical data correlation stability. Results are presented in Table 1 and illustrated in Figures 3 and 4. Equations used to calculate stability scores for numerical and categorical data are available in Multimedia Appendix 1.

**Table 1:** Fidelity scores and their components for the 5000 and 10,000 artificial patients generated.

Scores		5000 artificial patients, %	10,000 artificial patients, %
**Fidelity score**		97.8	94.6
	Numerical stability	100	100
	Categorical stability	96.4	91.2
	Numerical correlation stability	97.1	92.6

With fidelity scores of 97.8% and 94.6%, data of the 5000 and 10,000 artificial patients were considered realistic and representative of the real data.

To assess the confidentiality of newly generated artificial data, filter similarity scores (proportion of data not similar to the initial dataset) and degree of anonymization (Euclidean distance) were calculated. The results strongly confirm the nonsimilarity of the artificial data with the initial real data (filter similarity scores>99.9%) and a high degree of anonymization (the artificial data were further away from the initial dataset than the initial data were from each other).

### Plausibility of Artificial Patients

A categorization task was performed by 3 experienced anesthetists who were blinded, with the distribution of a balanced sample of 100 real and artificial patients generated using the VAE. Anesthetists were asked to determine whether each patient was real or artificial. The κ coefficients of agreement were –0.12 (95% CI –0.31 to 0.07), 0.15 (95% CI 0.1-0.26), and 0.09 (95% CI –0.14 to 0.15). Given the very low agreement coefficients (<0.2), none of the 3 experts could differentiate between real and artificial patients, arguing for the medical plausibility of the artificial patients.

## Discussion

Through this proof-of-concept study, we demonstrated, for the first time, the feasibility to transpose the VAE technique from imaging to HDLSS clinical data for the generation of a large number of artificial patients. The high fidelity scores obtained demonstrate the consistency of our artificial cohorts. Moreover, as suggested by the high filter similarity scores and the low agreement coefficients of the categorization task, the artificial patients could not be matched with real patients.

The use of artificial intelligence (AI) in health care presents particularly complex challenges inherent in the types of data it relies on (sensitive, sparse, heterogeneous, limited, etc). However, research efforts over the last few years, which have focused on meeting these specific challenges, now offer a glimpse of dizzying potential, particularly in the field of clinical research. In silico trials on artificial patient cohorts can quickly and cost-effectively include diverse minority groups (eg, rare diseases, children, pregnant women, and ethnic minorities), reducing risks and recruitment challenges of traditional in vivo trials. In this setting, artificial patients’ technology has the potential to transform health care by improving diagnosis, treatment, and mapping clinical pathways [3].

Validation of mathematical models and algorithms is important from an ethical point of view. Like any data processing, the implementation of artificial patient cohorts requires human oversight (in reference to the AI Act [10]). This principle of “Human Guarantee” refers to the need not to relinquish decision-making autonomy in the context of increasingly rapid dissemination of AI. Organized in the form of human oversight committees, these control measures will help better understand the phase of modeling artificial patient populations and ensure that it is as unbiased and reliable as possible. The need to protect health data and the rights of the individuals is a matter of debate that will likely be resolved with the expected advances in anonymization. Ensuring that artificial and real data are not similar is one way of guaranteeing anonymization and confidentiality. In this setting, no real patient should be identifiable from artificial data.

Our results must be interpreted with caution and a number of limitations should be borne in mind. First, our study presents promising results but does not present a comparative analysis with existing methods for generating artificial patient data (eg, GANs). In this setting, further studies designed to identify the most effective approach could be of major interest. Second, our study strongly lacks a comparative analysis with the late postoperative outcomes of real patients. Further studies integrating longitudinal dynamics are needed to map trends and identify patient trajectories. In this context, the recent update of MAX with the implementation of a postoperative digital conversational agent for the collection of recovery data based on patient-reported outcome measures could be of major interest. Indeed, we plan to compare longitudinal data from real patients with those obtained in a cohort of artificial patients. Finally, the results of our proof-of-concept study are encouraging, but it seems necessary to replicate the method. For instance, a process is underway to validate the reliability of artificial patient cohorts by replaying old clinical trials using data shared on the Yale University Open Data Access. Moreover, we plan to apply our VAE method to other kinds of data (eg, multimodal data). In this setting, the first European web platform for augmenting data and creating artificial patient cohorts with VAE’s generative AI has recently been created (ORIGA; BOTdesign).

To conclude, we demonstrate, for the first time, the feasibility to augment HDLSS clinical tabular data by using a VAE. The newly generated artificial patient cohorts were consistent with real source data. We believe that in silico trials can be used to track a variety of health indicators and generate key insights. Artificial patients will revolutionize health care paving the way for a more precise, personalized, and predictive medicine.

## Appendix

Multimedia Appendix 1 Additional material.

## Abbreviations

AI: artificial intelligence
GAN: generative adversarial network
HDLSS: high-dimension, low-sample-size
VAE: variational autoencoder

## Data Availability

Data are available from the corresponding author upon request.
