# Supplementary material for: Generating Artificial Patients With Reliable Clinical Characteristics Using a Geometry-Based Variational Autoencoder: Proof-of-Concept Feasibility Study
Source: J Med Internet Res. 2025 Apr 17;27:e63130. doi: 10.2196/63130 (PMC12046256; doi:10.2196/63130)
Supplement: Multimedia Appendix 1 [file jmir_v27i1e63130_app1.docx]

**SUPPLEMENTARY MATERIAL**

1. **TRAINING THE MODEL AND GENERATING ARTIFICIAL DATA**
2. **TRAINING THE MODEL**
   1. **Network architecture**

The neural network of the variational autoencoder (VAE) trained model was pre-implemented in the Pyraug module. The following figure illustrates its architecture.


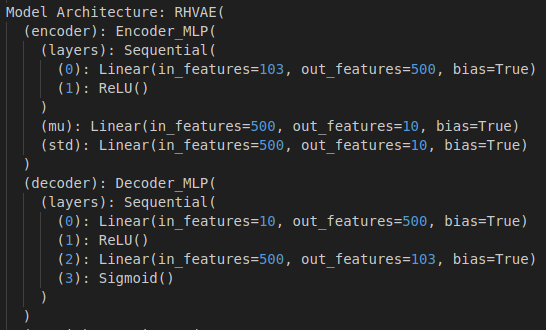


### Data pre-processing

The model was trained on a dataset comprising 521 patients (rows) and 85 variables (columns) extracted from the “MAX” digital conversational agent created for preparing patients for anesthesia (BOTdesign®, Toulouse, France).

Before being used for training, the dataset was pre-processed by the TabularDataProcessor. This produces a dataset of dimension (521, 103). This additional number of “columns” comes from variables that have undergone one-hot encoding. Note that among the categorical variables, we have chosen to encode some of them ordinally, to better reflect the hierarchy of available categories. Each of the following variables has had categories encoded in ascending numerical order:

- Mallampati: Class I, Class II, Class III, Class IV
- Mandibular mobility: Class I, Class II, Class III
- Metabolic Equivalent Task: ‘Toileting / Eating alone / Walking around the home / Able to walk 100 m flat’, ‘Climbing 2 flights of stairs / Walking on a steep incline / Intensive gardening / Moving furniture’, ‘Intense / Swimming / Cycling etc’
- Cigarettes number: 0, 1 to 5, 6 to 10, 11 to 15, 16 to 20, 21 to 30, over 30,
- Past intervention (number): 1, 2, 3, 4, 5, 6, 7, 8, 9, 10, over 10

Each variable also underwent a scaling of its values between 0 and 1.

### Training parameters

The model was trained for 1000 epochs, with a batch size of 32 and a learning rate of 0.001. Actually, training was stopped earlier (at iteration 945), due to the absence of any noticeable improvement (loss). Again, this early stop has been pre-implemented in Pyraug as it prevents overlearning. The following figure illustrates loss evolution during model training.


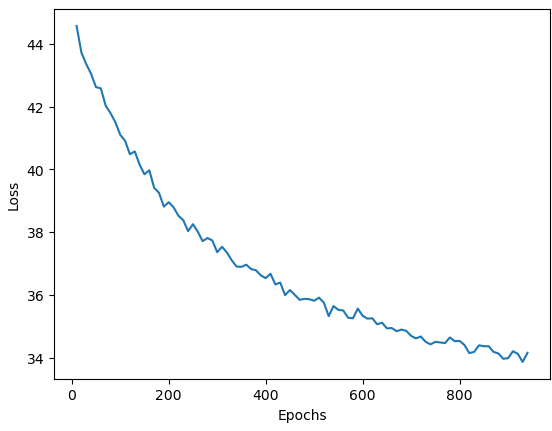


1. **GENERATING ARTIFICIAL DATA**

Based on the previously trained model, 2 artificial datasets were generated. The first with 5,000 artificial patients, the second with 10,000 artificial patients. These artificial datasets respectively represent a data augmentation ratio of almost 10 and 20 artificial patients for 1 real patient (521 patients in the initial dataset).

The data generated by the model had to be post-processed in order to be usable, i.e., they must undergo the reverse processing to that which allowed them to be ingested by the model. This is done using the TabularDataProcessor and the JSON file associated with the training.

The resulting dataset thus had the same formatting as the original data. In this setting, no further post-processing was carried out. Overall, the model respects the dependencies between certain variables (e.g., gender (M/F) and postpartum bleeding (Yes/No), or smoking (Yes/No) and cigarettes number).

1. **STABILITY SCORES**
2. **Distribution stability score of numerical data**

To ensure the stability of the numerical data structures, the non-parametric Kolmogorov-Smirnov statistical test was used to compare two continuous distributions.

1. **Distribution stability score of categorical data**

The stability score of categorical data distribution is described with the following equation:

$$S =1 -\frac{1}{|C|} \sum_{v in V} \sum_{c in v} |s_{c}-r_{c}|$$

With:

- $S$denoting the stability score of the categorical data distribution

- $V$ representing the set of categorical variables

- $v$ designating a categorical variable

- $c$ designating a class in the categorical variable $v$

- $s_{c}$ designating the proposition of the $c$ class in the categorical variable $v$ in the artificial dataset

- $r_{c}$ designating the proposition of the $c$ class in the categorical variable $v$ in the real dataset.

- $|C|$ designating the total number of classes present in the dataset.

1. **Correlation stability score of numerical data**

The stability score of numerical data correlation is described with the following equation:

$$S =1 - \frac{1}{|V|} \sum_{v in V} |s_{c}-r_{c}|$$

With:

- $S$denoting the stability score of the numerical data correlation

- $V$ representing the set of numerical variables

- $v$ designating a numerical variable

- $s_{c}$ designating the $c$ class proposition in the $v$ numerical variable in the artificial dataset

- $r_{c}$ designating the $c$ class proposition in the $v$ numerical variable in the real dataset
